# Mutations in a Conserved Domain of *E*. *coli* MscS to the Most Conserved Superfamily Residue Leads to Kinetic Changes

**DOI:** 10.1371/journal.pone.0136756

**Published:** 2015-09-04

**Authors:** Hannah R. Malcolm, Paul Blount

**Affiliations:** Department of Physiology, University of Texas Southwestern Medical Center, Dallas, Texas, 76390, United States of America; Zhejiang University, CHINA

## Abstract

In *Escherichia coli* (*E*. *coli*) the mechanosensitive channel of small conductance, MscS, gates in response to membrane tension created from acute external hypoosmotic shock, thus rescuing the bacterium from cell lysis. *E*. *coli* MscS is the most well studied member of the MscS superfamily of channels, whose members are found throughout the bacterial and plant kingdoms. Homology to the pore lining helix and upper vestibule domain of *E*. *coli* MscS is required for inclusion into the superfamily. Although highly conserved, in the second half of the pore lining helix (TM3B), *E*. *coli* MscS has five residues significantly different from other members of the superfamily. In superfamilies such as this, it remains unclear why variations within such a homologous region occur: is it tolerance of alternate residues, or does it define functional variance within the superfamily? Point mutations (S114I/T, L118F, A120S, L123F, F127E/K/T) and patch clamp electrophysiology were used to study the effect of changing these residues in *E*. *coli* MscS on sensitivity and gating. The data indicate that variation at these locations do not consistently lead to wildtype channel phenotypes, nor do they define large changes in mechanosensation, but often appear to effect changes in the *E*. *coli* MscS channel gating kinetics.

## Introduction

Proteins are often classified into homology groups, or families, based on high sequence homology or similar functional roles (functional homologues). For functional homologues, these family members often have diverse sequences but all complete the same function within the cell. However, in protein families where members are identified based on sequence homology to a particular region, the functional roles of these proteins has the potential to be quite diverse. It remains unclear why variations within such a homologous region occur: is it tolerance of alternate residues, or does it define functional variance within the superfamily? In the mechanosensitive channel of small conductance (MscS) superfamily of channels, members are identified by homology to a highly conserved region of approximately 90 amino acids in the pore lining helix and the upper vestibule domain; outside of this region, although the sequence homology is significantly diminished, there is still predicted to be some structural homology throughout the vestibule domain to *Escherichia coli* (*E*. *coli*) MscS (Ec-MscS) [1–3]. The 15 subfamilies of the MscS superfamily are unique due to additions on either the N-terminus or C-terminus to the family root channel Ec-MscS, such as additional transmembrane domains, ligand binding domains, large extracellular loops, and in some cases a domain that is predicted to interact with the outer membrane.

Ec-MscS functions as a pressure relief valve, releasing water and osmolytes in response to hypoosmotic shock [4–8]. It appears to gate in response to tension within the membrane in a manner similar to a Jack-in-the-Box, springing into the open state when the applied extrinsic tension overcomes the intrinsic bilayer tension [9]. The *E*. *coli* genome has seven mechanosensitive channels: six MscS homologues[10, 11] and the mechanosensitive channel of large conductance (MscL), a non-related mechanosensitive channel that gates just prior to the lytic tension[12, 13]. Channels that are predicted to be homologous to Ec-MscS have been identified in essentially all bacterial, many plant, and some yeast genomes; in the majority of these genomes multiple MscS superfamily members have been identified [14–19].

**Fig 1:**
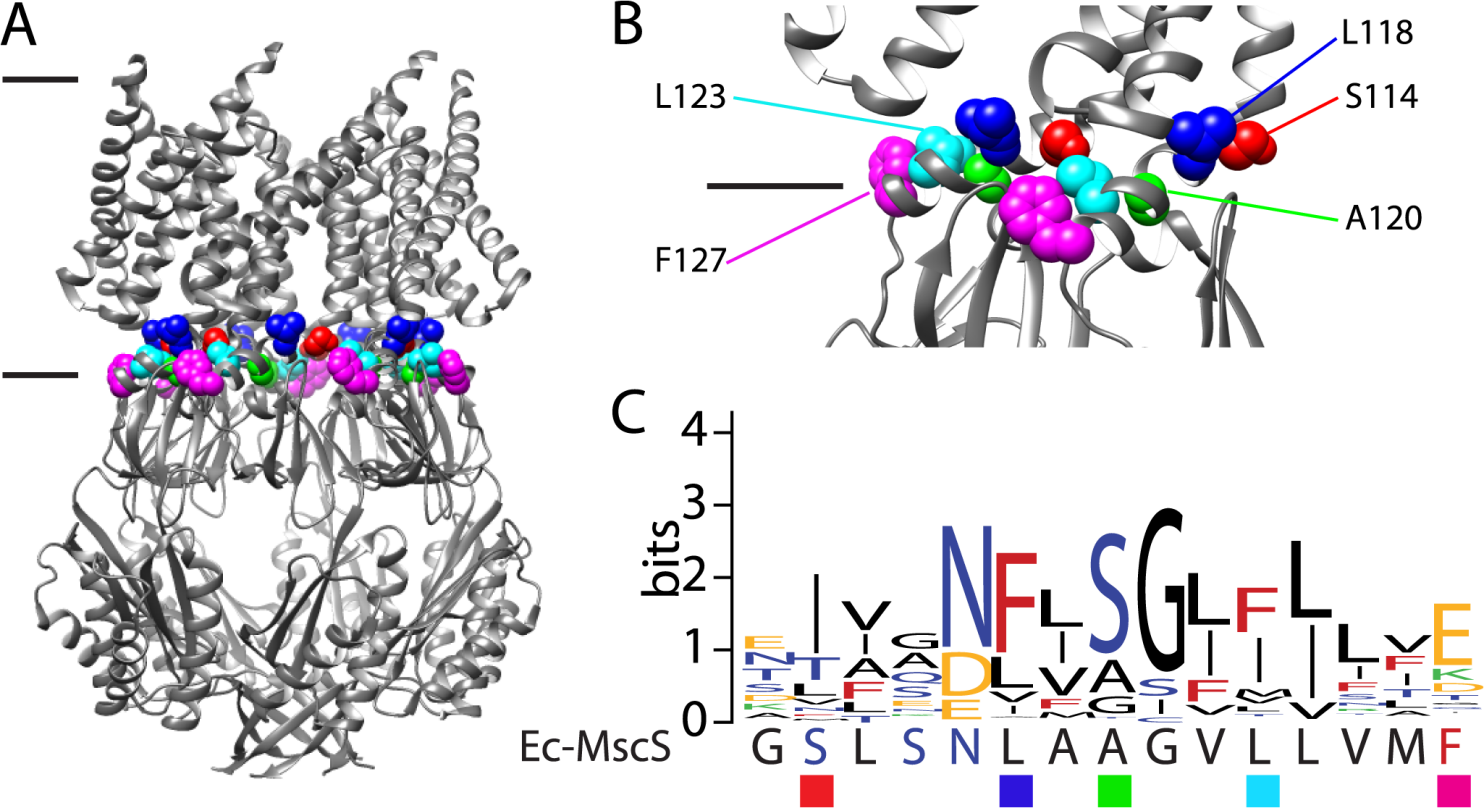
Conservation in TM3B in Ec-MscS. A) Structural representation of the five residues (S114, red; L118, blue; A120, lime green; L123, aqua; F127, magenta) on the open state crystal structure, black lines indicate the predicted location of the lipid headgroups. B) A close-up of the location of these residues is shown, for clarity only two adjacent subunits are shown. C) A conservation map of TM3B comparing the amino acid sequence of Ec-MscS with the residues of highest conservation within the MscS superfamily. Larger amino acids indicate higher conservation at that residue, the y-axis in bits gives the maximum sequence conservation, log_2_(20) = 4.13. Hydrophobic residues (I, P, L, M, V, A, G), are colored black; aromatic residues (F, W, Y) are colored red; polar residues (S, T, Q, N, C) are colored blue; basic residues (K, R, H) are colored green; and acidic residues (D, E) are colored yellow. *E*. *coli* MscS residues that are significantly different from the conserved residue are indicated with a ■ below, in colors corresponding to the structural representation.

Several crystal structures of the full-length Ec-MscS exist reflecting multiple states[20–24]. Such models predict that each subunit contains three transmembrane domains and that the complex is a homo-heptamer, with a large vestibule or “cage” domain residing within the cell cytoplasm (Fig 1A). Ions travel out of the cell through the 8–12Å pore formed by the pore lining helix, TM3. The pore lining helix is composed of two parts, TM3A and TM3B, connected by a hinge at G113[25, 26]. TM3B is predicted to be involved in structural stabilization of the inactivated state through protein-protein interactions with the ß-domain [27]. Additionally, Rowe et. al. show that the interactions between TM3B and the ß-domain are involved in the inactivated state and that destabilization of these interactions prevent entry to the inactivated state [28]. This wealth of structural models has given significant insight into the movement of the transmembrane domains throughout the gating cycle of MscS, specifically a static picture of the starting and ending points. It has been predicted that the structural movements that Ec-MscS undergoes in the gating process are conserved throughout the MscS superfamily [29, 30].

Previous electrophysiological studies on the gating of MscS in response to tension have shown that the wildtype channel opens in response to tension applied in the membrane. A very rapid inactivation of Ec-MscS is observed in excised patches from spheroplast membranes that are patched at low pH (e.g.: 6.0) [31]. At neutral pH this rapid inactivation is not observed, however time-dependent inactivation is observed as well as inactivation upon prolonged sub-threshold pressure application [32–34]. However, studies on several of the MscS superfamily subfamilies suggest that not all channels function the same way. For some of the subfamilies, mechanosensitivity has not been observed, suggesting either that the channel has evolved to gate in response to a different stimuli or set of stimuli, or that other factors are required to gate in response to mechanical tension[1, 29]. In some cases, slight mechanosensation has been observed in non-mechanosensitive superfamily members that normally have numerous extraneous domains, but have been pared down to a channel that resembles Ec-MscS[29].

Analysis of a homology plot of the superfamily, as performed previously by Malcolm and Maurer [1], which is consistent with the homology analysis conducted in [35], shows significant differences when compared to the sequence of Ec-MscS, particularly in the conserved TM3B region (Fig 1B and 1C). In the homology plot in Fig 1C the height of the letter corresponds to the degree at which it is conserved in the 100 plus sequences used to generate the homology plot [1]. The letters are color coded to correspond to the general class of amino acid (ie: black for hydrophobic, yellow for acidic residues, see the figure caption for remaining classes), below the homology plot the corresponding residues for Ec-MscS are shown for comparison. The most significant difference are observed for residues S114, L118, A120, L123, and F127, where the residues observed for Ec-MscS are significantly different, ie: for L123 the hydrophobic amino acid is replaced with an aromatic residue. This region connects the pore lining helix and the upper vestibule domain and is predicted to move when the channel springs into the open state. To study how the most significant of these alterations within the high homology region affects Ec-MscS gating, point mutations (S114I/T, L118F, A120S, L123F, F127E/K/T) and patch clamp electrophysiology were utilized. In this study we determine that while these changes do not usually affect the ability of Ec-MscS to respond to tension in the membrane, the kinetics of channel opening can be greatly altered. These alterations to channel kinetics are likely due to the destabilization of the normally stable open state, sometimes rapidly going into an inactivated state for some mutated channels. Moreover, these data suggest that a single point mutation can drastically alter the response of Ec-MscS to prolonged tension in the membrane. Thus, variations within this conserved region of the protein, and their protein-protein, and possibly protein-lipid interactions, may be one factor defining subtle changes in kinetics and inactivation.

## Materials and Methods

### Strains and Plasmids

Mutational cloning was conducted using the DH5α *E*. *coli* strain. All mutated channels were C-terminal six-His tagged and subcloned into the pB10d [36] vector with a LacUV5 promoter. Patch clamp electrophysiology was conducted in MJF429 (*MscS* and *MscK* null) [4].

### Site-Directed Mutagenesis

Ec-MscS residues that are unique from the MscS superfamily conservation residues were mutated to the most conserved residue using Megaprimer mutagenesis [37]. All mutations in MscS were cloned into the pB10d vector. For Megaprimer mutagenesis, forward and reverse primers located in the vector were used as the exterior primers and primers for mutagenesis were designed using Stratagene’s QuikChange Primer design tool. Mutations were verified by enzymatic digestion and sequences confirmed using automated sequencing (Big Dye v3.1, Applied Biosystems, Carlsbad, CA).

### Spheroplast Preparation

*E*. *coli* spheroplasts of MJF429 cells containing the indicated pB10d constructs were prepared as previously described [31]. Briefly, cells were grown in LB containing ampicillin and 0.06 mg/mL Cephalexin at 37°C with shaking until filamentous cells were approximately 50–150 μm. Cells were induced with 1 mM IPTG for 5 minutes and then harvested at 2500 x g (expression was conducted for only 5 minutes to keep channel expression low for electrophysiological analysis as previously described [1, 38]). Pellets were resuspended in 2.5 mL 0.8 M sucrose prior to sequential addition of 125 μL 1 M Tris pH 8.0, 120 μL 5 mg/mL Lysozyme, 30 μL 5 mg/mL DNase, and 150 μL 125 mM EDTA at pH 7.8. Lysozyme digestion proceeded for 5 minutes prior to the addition of 1 mL stop solution (0.67 M Sucrose, 19.4 mM MgCl_2_, 9.7 mM Tris at pH 8.0). The reaction mixture was layered over 7 mL chilled dilution solution (0.8 M Sucrose, 10 mM MgCl_2_, 10 mM Tris at pH 8.0) in test tubes. Spheroplasts were pelleted by spinning at 1600 x g for 2 minutes at 4°C and the pellets were gently resuspended in the dilution solution and stored in aliquots at-20°C.

### Patch Clamp Electrophysiology

Excised, inside out patches from *E*. *coli* giant spheroplasts were studied at room temperature as previously described [39]. Patch buffer contained 200 mM KCl, 90 mM MgCl_2_, 10 mM CaCl_2_, 5 mM HEPES at pH 7.5. Data was acquired using an AxoPatch 200B amplifier in conjunction with Clampex 10.3 (Molecular Devices) at-20mV and a sampling rate of 30303 Hz, with a 5 kHz lowpass filter. The pressure applied to the patch throughout the experiment was monitored with a piezoelectric pressure transducer (WPI). Traces were analyzed using Clampfit 10.3 (Molecular Devices) using the decay rate kinetics tool and the open dwell time tool. The open dwell time was calculated for at least 200 channels from multiple patches. The *E*. *coli* mechanosensitive channel of large conductance (MscL) was used as the internal standard for determining the pressure thresholds, as previously described [40–42]. Utilizing *E*. *coli* MscL as the internal standard for patch clamp electrophysiology rectifies any difference in the pressure required to open the channels due to alterations in patch geometry (in accordance with La Place’s law) as both channels sense tension in the membrane, and thus will be equally effected by the patch geometry. Pressure thresholds were obtained by dividing the pressure at which the second MscS opens by the pressure at which the first MscL opens (P_S_/P_L_) [43]. P_S_/P_L_ ratios were calculated from at least four patches from a minimum of two independent spheroplast preparations for each mutation. To study channel kinetics for these channels, tension was applied to the patch until a maximal number of channels opened, the tension was held constant for 4–8 seconds to observe the channel kinetics, and then additional pressure was applied to open MscL.

## Results

### Selection of Point Mutations

Members of the MscS superfamily of ion channels are identified by significant homology to an 87 amino acid region in Ec-MscS that starts at the pore lining helix and extends through the upper vestibule domain [1, 16, 17]. When comparing the highest conserved residues with the Ec-MscS sequences in the TM3B region, significant differences were observed for five residues: S114, L118, A120, L123, and F127 (Fig 1B). These residues are located on the C-terminal end of the pore lining helix and are predicted to move in the transition between the closed and open states. To determine if these residues are involved in the ability of Ec-MscS to gate in response to the relief of lateral tension in the membrane, point mutations were generated. Residues in Ec-MscS were mutated to the highest conserved residue from the MscS superfamily: S114 to S114I/T, L118 to L118F, A120 to A120S, L123 to L123F, and F127 to F127E/K/T. Channels with these point mutations were studied with patch clamp electrophysiology to understand their influence on MscS function.

### Patch Clamp Electrophysiology of Channels with Point Mutations

#### Mechanosensitivity of Point Mutations

**Fig 2:**
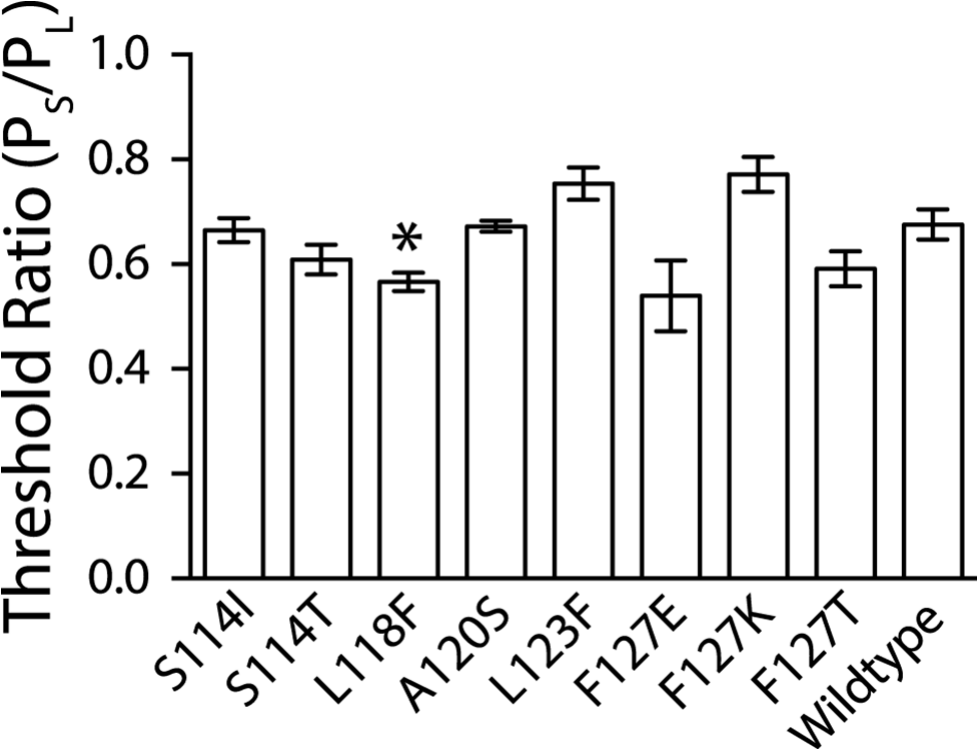
Pressure threshold ratios for Ec-MscS with single point mutations at the sites indicated, to the most conserved residue in the MscS Superfamily. Error bars represent the standard error of the mean for a minimum of 4 patches from a minimum of two independent preps. The star indicates the construct that is statistically different from wild-type MscS as determined using a Student’s T-test (* = p < 0.05).

Patch clamp electrophysiology experiments were conducted in the *E*. *coli* strain MJF429 (null for MscS and MscK, a potassium dependent mechanosensitive channel that has a similar conductance to MscS) [4], so that MscS-like activity was absent, yet the *E*. *coli* mechanosensitive channel of large conductance (MscL) could be utilized as an internal standard. *E*. *coli* MscL gates in response to tension just prior to cell lysis, at significantly higher membrane tensions than that required to gate Ec-MscS[44]. To utilize *E*. *coli* MscL as an internal control, the pressure at which the second MscS (P_S_) opens is divided by the pressure at which the first MscL opens (P_L_), giving a threshold ratio (P_S_/P_L_) [43]. These threshold ratios allow the response of each mutated channel to be compared to wildtype MscS as well as other mutants by accounting for patch to patch variation. To determine how these mutated channels responded to applied tension in the membrane, pressure threshold ratios were determined (Fig 2). None of the studied point mutations showed a large difference in pressure threshold in comparison to the wildtype threshold. However, L118F showed a very slight but significant difference from wildtype, as calculated with an unpaired Students T-test (p<0.05), gating at a statistically lower pressure thresholds, thus identifying it as having a very slight gain of function phenotype. The other seven mutations were determined not to be statistically significantly different from wildtype. To identify the channel kinetics, tension was applied to the patch until a maximal number of channels opened (normally 10 to 30), the tension was held constant for 4–8 seconds to observe the channel kinetics, and then additional pressure was applied to open MscL. When membrane tension was applied to each of the patches in this manner, significant differences in the channel kinetics were observed in a subset of channels.

#### Kinetic Analysis of Point Mutations

**Fig 3:**
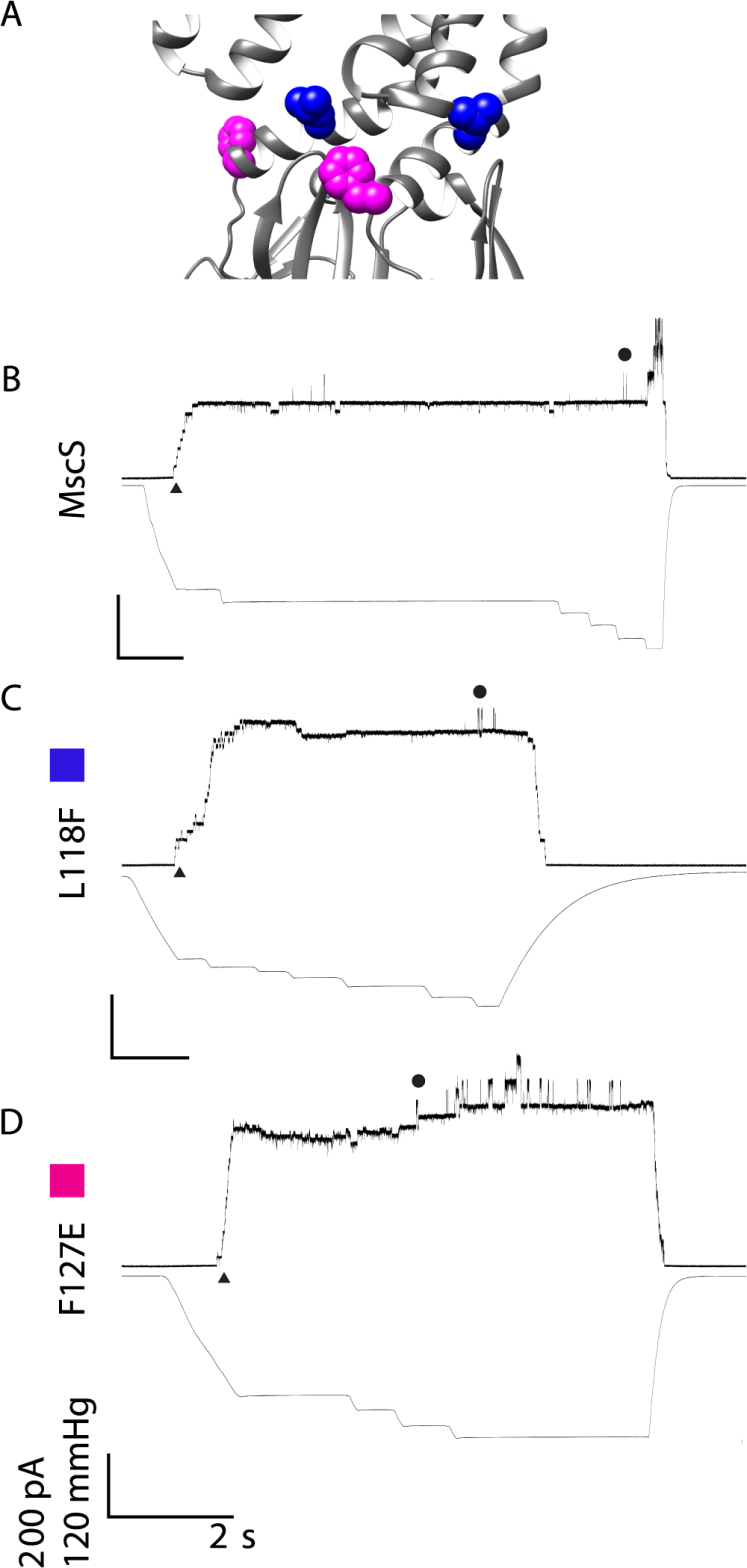
Representative traces are shown for wildtype MscS and mutated channels that show kinetically wildtype behavior. A) Structural Representation of residues of interest B) Wildtype MscS C) L118F D) F127E The representative traces show the kinetic phenotype observed for the mutated channels in all patches from at least two independent spheroplast preparations. For simplicity, channel openings are shown as upward inflections, MscS openings are indicated by ▲ and MscL openings are indicated with ●. Scale bars represent 2 seconds on the X-axis and 200 pA and 120 mmHg on the Y-axis. Traces are labeled with a color box corresponding to the color in the structural representation for clarity.

To further study the kinetics of these channels several kinetic analyses were calculated: single channel conductance, the rate of inactivation, and the open dwell time (Table 1). Based on the rate at which the channels closed, the mutated channels fell into three easily definable categories: kinetically similar to wildtype, having inactivation rates that are less than 4 seconds, and having a shortened open dwell time, thus flickering between open and closed states. For wildtype Ec-MscS, the channels open in a stair-step manner upon the negative pressure ramp and remain open at a relatively consistent level prior to the opening of MscL (Fig 3). The conductance of the wildtype channel was determined to be 1.32±0.08 nS. Many groups have observed this classical gating manner when Ec-MscS is studied at pH 7.2–7.5 [9, 26, 34, 45].

**Table 1:** Kinetic values for wildtype channel and mutants.

	Pressure Threshold Ratio (P_S_/P_L_)	Conductance (nS)	Rate of Inactivation (sec)	Open Dwell Time (msec)
WT	0.69±0.03	1.32±0.08	ND^1^	ND^2^
L118F	0.56±0.02	1.34±0.06	ND^1^	ND^2^
F127E	0.54±0.07	1.33±0.04	ND^1^	ND^2^
A120S	0.67±0.01	1.30±0.02	2.1±1.5	ND^2^
F127T	0.59±0.03	1.36±0.08	3.0±1.8	ND^2^
S114I	0.65±0.02	0.96±0.08	1.4±0.1	1.4±1.7
S114T	0.61±0.03	1.34±0.04	1.9±0.5	2.1±3.6
L123F	0.73±0.03	1.25±0.04	ND^1^	0.8±0.9
F127K	0.77±0.03	1.33±0.04	0.6±0.2	2.0±2.9

All values have been calculated from at least 4 independent patches from at least two preps and the error is the standard deviation.

ND^1^: For some mutants the rate of inactivation was infinity meaning that in the time that the pressure was applied no inactivation was observed.

ND^2^: Some channels did not have discrete single channel openings because of long open dwell times, and thus the open dwell time for the channels were not calculated.

#### Electrophysiology of Point Mutations with Wildtype Kinetics

L118F and F127E both have kinetic phenotypes that are indistinguishable from Ec-MscS (Fig 3). As negative pressure is ramped, these channels open in the classical stair-step manner prior to reaching the full and stable open state until the pressure threshold for MscL is achieved. Analyses demonstrate that both of these channels have a conductance similar to the wildtype channel (1.34±0.06 nS and 1.33±0.04 nS respectively). Additionally, these channels do not inactivate or have a short open dwell time (Table 1). These data suggest that some amino acid size alteration is tolerated at these sites and that these mutations are not detrimental to channel activity.

#### Electrophysiology of Point Mutations with Rapidly Inactivating Kinetics

**Fig 4:**
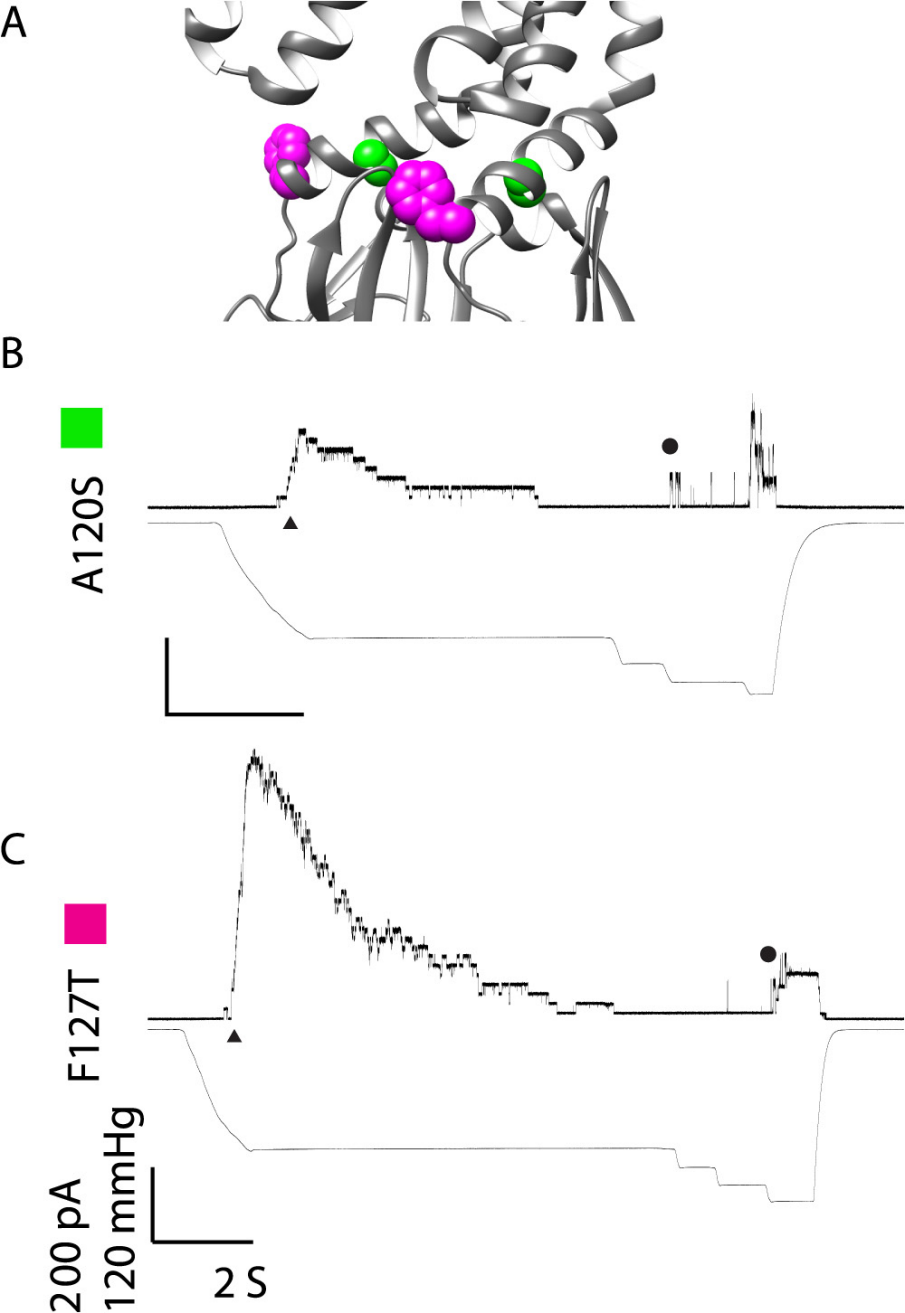
Representative traces are shown for mutated channels that rapidly inactivate upon pressure application. A) Structural Representation of residues of interest B) A120S C) F127T The representative traces show the kinetic phenotype observed for the mutated channels in all patches from at least two independent spheroplast preparations. For simplicity, channel openings are shown as upward inflections, MscS openings are indicated by ▲ and MscL openings are indicated with ●. Scale bars represent 2 seconds on the X-axis and 200 pA and 120 mmHg on the Y-axis. Traces are labeled with a color box corresponding to the color in the structural representation for clarity.

A120S and F127T are kinetically different from Ec-MscS in that after the classical stair-step openings, the channels inactivated within 2–4 seconds (Table 1 and Fig 4; note that the latter uses the same scale bars as Fig 3 so direct comparisons can be made). This behavior was observed in all patches for these mutations (4 patches from at least 2 independent preps). While this sort of inactivation is observed at low pH for the Ec-MscS channel (e.g. pH 6.0), it is never seen at the neutral pH used in these experiments [31]. The conductance for these channels, however, is indistinguishable from wildtype (1.30±0.02 nS and 1.36±0.08 nS respectively). Hence, the only deficit of these channels appears to be that they transition rapidly from the open state to an inactivated state.

#### Electrophysiology of Point Mutations with Short Open Dwell Times

**Fig 5:**
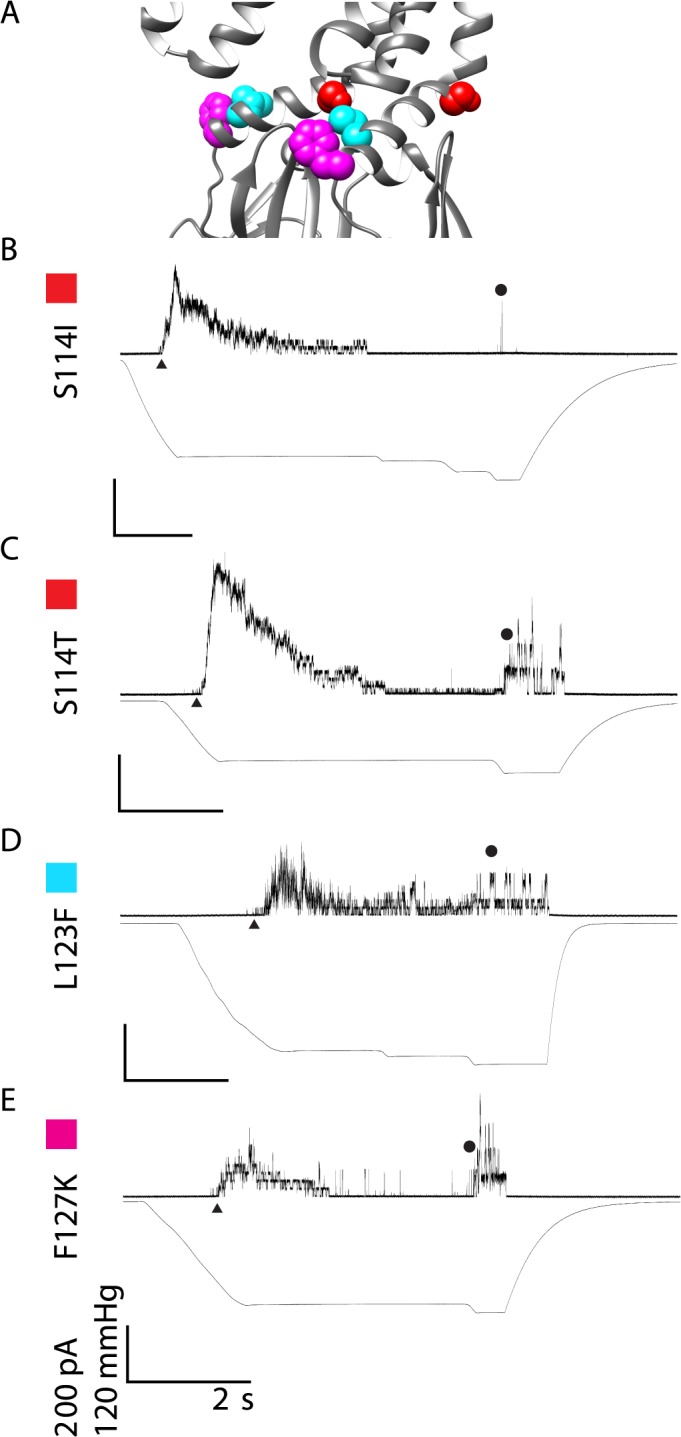
Representative traces are shown for mutated channels that have a destabilized open state upon pressure application. A) Structural Representation of residues of interest B) S114I C) S114T D) L123F E) F127K The representative traces show the kinetic phenotype observed for the mutated channels in all patches from at least two independent spheroplast preparations. For simplicity, channel openings are shown as upward inflections, MscS openings are indicated by ▲ and MscL openings are indicated with ●. Scale bars represent 2 seconds on the X-axis and 200 pA and 120 mmHg on the Y-axis. Traces are labeled with a color box corresponding to the color in the structural representation for clarity.

S114I, S114T, L123F, and F127K are kinetically different from Ec-MscS in that they appear to flicker open and closed having average open dwell times that are less than 3 ms (Fig 5; note that the scale bars are the same as Figs 3 and 4 so direct comparisons can be made). Both of the S114 mutations and F127K rapidly inactivate when the tension is held steady. The conductances of the majority of the channels, S114T, L123F, and F127K, are not statistically significantly different from wildtype and are 1.34±0.04 nS, 1.25±0.04 nS, and 1.33±0.04 nS respectively. However, S114I shows a slight reduction in channel conductance (0.96±0.08 nS compared to wildtype 1.32±0.08 nS) that is statistically significant using an unpaired Students T-test (p<0.05). While S114I can achieve close to the full open state, either the rapid inactivation of these channels, or the destabilization of the protein-protein contacts that normally occur upon opening, prevent the channel from reaching a uniform and stable fully open state. L123F constantly opens and closes when tension is applied to the membrane, making the channel appear to flicker back and forth between the open and closed states. Additionally, the unstable openings of L123F can obscure the MscL openings when several MscS L123F channels open at the same time. More than likely this flickering phenotype is due to a destabilization of the full open state of MscS; however, we observed that the amount of tension required to open these channels has not changed. It is of note that unlike the other channels displaying a short open dwell time, L123F does not inactivate in response to pressure, demonstrating that rapid dwell times and inactivation are not coupled.

## Discussion

### General Analysis of Mutations

Mutations to the most conserved residue within the MscS superfamily in the TM3B domain of Ec-MscS lead to significant kinetic changes. The channel activities are observed to be functionally wildtype, channels that inactivate within 2–4 seconds, or flickering between the open and closed states. In the case of F127 all three kinetic phenotypes were observed in response to the different point mutations. Together these residues appear to be involved in the stability of the open state of MscS through protein-protein packing interactions that are easily disturbed by point mutations. Because it is likely that each of the five residues plays a unique role in the gating cycle of Ec-MscS, each is discussed separately, below.

### S114

**Fig 6:**
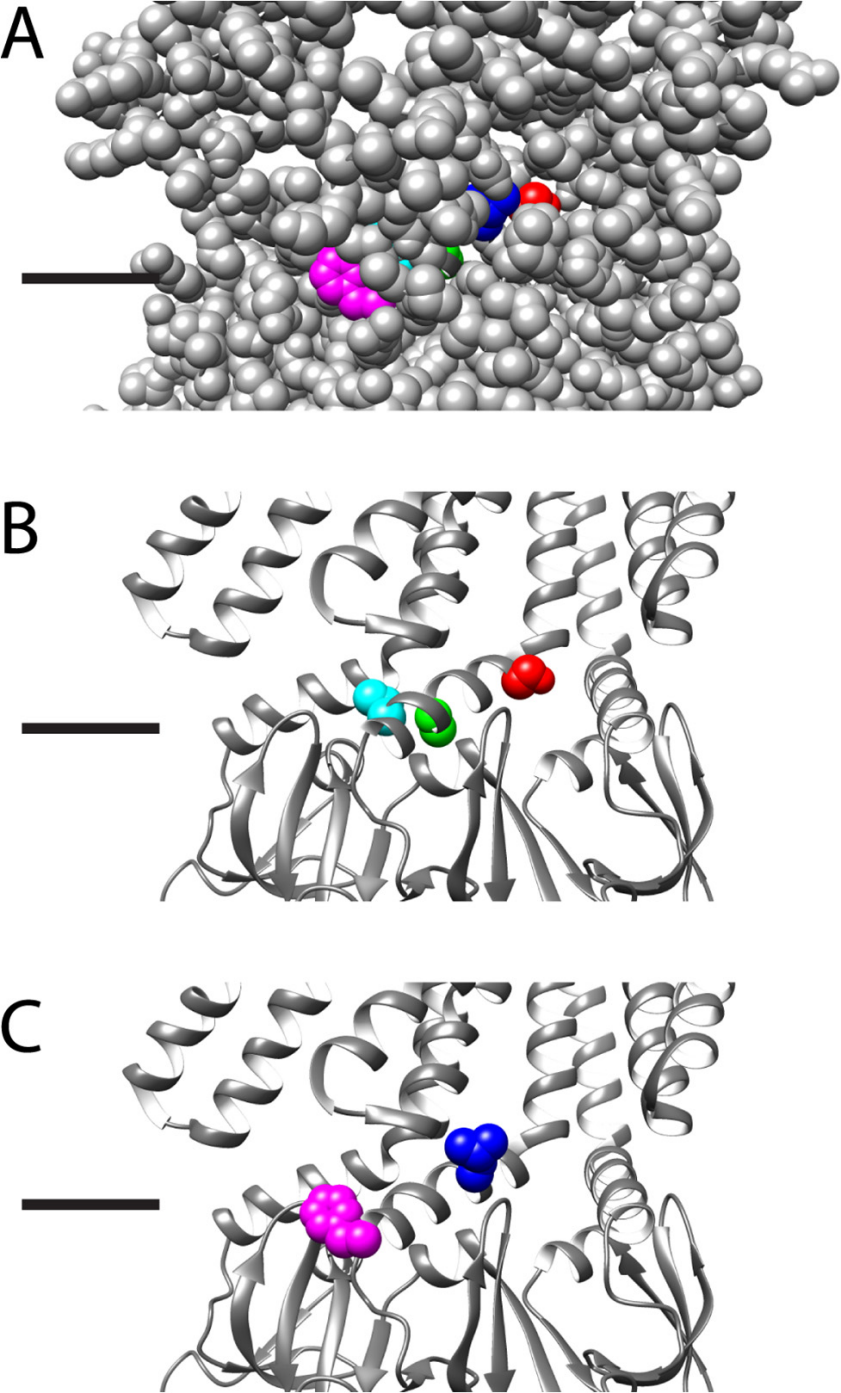
Structural representation of the five residues on the open state crystal structure. Structural representation of the residues (S114, red; L118, blue; A120, lime green; L123, aqua; F127, magenta) on the open state crystal structure, black lines indicate the predicted location of the lipid headgroups, for clarity three adjacent subunits are shown and the residues are shown as spacefilling in color on the center subunit only. A) All residues are shown as spacefill, showing that L118 and F127 are “lipid exposed”, or visible from the exterior of the protein, while S114, A120, and L123 are buried. B) The protein backbone is shown as a ribbon, and the buried residues are shown in CPK. These residues when mutated are predicted to alter the protein-protein interactions leading to kinetic changes. C) The protein backbone is shown as a ribbon, and the lipid-exposed residues are shown in CPK. As these residues are predicted to be on the surface of the protein it is likely that their interactions with the lipids lead to alteration of channel behavior.

S114 is not exposed to the exterior surface, and therefore buried, potentially involved in protein-protein interactions (Fig 6A and 6B). In addition, this residue is adjacent to the first glycine kink, at G113, in the pore lining helix, which is known to play a role in gating kinetics [25, 26]. The two mutations at S114 cause the channels to rapidly inactivate as well as have a significantly shorter open dwell times, giving the appearance of a channel that flickers between the open and closed state prior to inactivation (Fig 5). The S114T mutation is conventionally classified as a conserved mutation that should allow for similar interactions to occur. However, S114T rapidly inactivates and flickers, suggesting that the addition of the methylene group to the serine, in the threonine, perturbs both the packing of TM3B as well as any potential interactions that the hydroxyl group might have with another amino acid. S114I shows similar kinetic behavior to S114T, suggesting that the addition of a significantly bulkier amino acid also perturbs the packing of TM3B. S114I shows a slight reduction in channel conductance, approximately 30% reduction in comparison to the wild-type channel. However, analysis of single channel openings shows that S114I does occasionally achieve a near to fully open amplitude but the majority of the gating events occupy an open substate. Together, these two point mutations show that within this region even small changes, such as S114T, lead to great kinetic changes that can in part be attributed to disturbing the tightly packed region just posterior to the channel pore. As S114 is distal to the pore, the decrease in conductance for S114I is potentially due to the additional bulk of the isoleucine preventing some of the twisting motion that TM3 is predicted to undergo during the gating cycle.

Previous work has shown that by mutating a glycine to an alanine, thus adding a single methylene group to G113, prevents the channel from inactivating when tension is applied to the patch, presumably because this change inhibits a pivot necessary for achieving this state [25, 26]. It is highly likely that one of the regions that are disturbed in the S114 mutations is this G113 kink, as the added bulk at this position may prevent or enhance the kink. The data derived from these mutated channels suggest that this kink is sensitive to changes within the region that disturb the protein-protein packing throughout TM3B. The additional bulk of these two mutations, instead of leading to a channel that does not inactivate, leads to channels that rapidly inactivate by disturbing the packing interactions, and perhaps twisting motions that the G113 is involved in, that are essential for the stable open state.

### L118

The substitution of a significantly larger aromatic residue for L118 does not functionally change the gating kinetics of Ec-MscS (Fig 3). The channels open upon the application of tension and achieve the stable open state, with no inactivation observed prior to MscL opening at a higher tension. L118F gates at a slightly lower pressure threshold than wildtype MscS, identifying this channel as having a gain of function phenotype. While L118 is not predicted to interact directly with the lipids (see Fig 6A and 6C), it does appear to be near the surface of the protein and the addition of the aromatic ring presumably disturbs the protein-protein packing in TM3B as well as adjusting the lateral tension applied to the membrane. The addition of the phenyl ring, most likely through long-range conformational changes, presumably disturbs the lateral tension applied by the lipids, thus allowing the channel to more easily spring into the open state. This alteration to the intrinsic lipid tension does not affect the channel once it opens, it merely reduces the activation energy from the closed to the open state, allowing the channel to open more easily.

### A120

The mutation of A120 to a serine leads to a channel that has relatively normal opening kinetics, but inactivates instead of achieving the stable open state (Fig 4). Serine is significantly larger than alanine and has a hydroxyl group; both of these factors most likely contribute to the destabilization of the stable open state. As the conductance of this mutation is not altered and the ability of the channel to gate in response to tension applied in the membrane is not disturbed, the additional bulk to the residue probably disrupts the tight packing that is essential for the stable open state (Fig 6A and 6B). Previous work on A120, utilizing an A120G mutation, suggests that alterations close to or at G121 alters the G121 kink structure, which can either prevent the entrance to the inactivated state or propel the channel into the inactivated state [25]. Taken together the work on A120S and A120G strongly suggest that alterations to the G121 kink lead to channels that rapidly inactivate due to alterations in the protein-protein packing that A120 is involved with or by altering the helical structure adjacent to the G121 kink.

### L123

When an aromatic ring is substituted for the large hydrophobic residue at L123 the stable open state is destabilized, allowing the channel to flicker between the open and closed state (Fig 5). Based on the open state crystal structure, although L123 is not exposed to lipids, L123 and F127 are on the same helical face of TM3B (Figs 1B and 6A), thus the addition of a phenyl ring one turn earlier most likely disturbs the role of F127 in the open state, leading to the destabilization observed. L123F does not appear to inactivate at any point but continuously flickers between the two states as the tension in the membrane is applied. The addition of the aromatic phenyl ring does not disturb the conductance of the open state, just the stability of the open state.

### F127

All of the point mutations made at F127 show drastically different phenotypes: wildtype, inactivation within 2–4 seconds, and flickering between the closed and open states (Figs 3, 4, and 5). The observation of significantly different phenotypes suggests that F127 is involved in the stabilization of the open state and has significant movement while the channel is opening. A significant point of interest is that the oppositely charged mutations show different phenotypes; the negative residue F127E is kinetically wildtype while the positive residue F127K is a slight loss of function that rapidly inactivates. This dichotomy suggests that F127 is involved in a long-range interaction with a full or partial positive charge that could be within the lipid headgroups or an as yet unidentified residue elsewhere within the protein (Fig 6A and 6C). By this notion, the addition of a positive residue creates a positive-positive repulsion, which would thus lead to the slight loss of function phenotype, while the addition of a negative charge at F127 leads to a charge-charge stabilization that compensates for the lack of an aromatic ring. The F127T mutation, which rapidly inactivates with a relatively normal kinetic phenotype when the channel opens, shows that the phenyl-ring is involved in the stabilization of the open state.

The long-range protein-protein interaction of F127 is not readily apparent on the Ec-MscS crystal structures predicted to be in the open or desensitized states [20, 23, 24]. However, this interaction is supported by the electrophysiological data, and future studies will investigate this interaction. The possibility of this long-range interaction being with the lipid headgroups is supported by electron paramagnetic resonance (EPR) data that shows F127 is in close proximity to the lipid headgroups in the closed state [21]. Additionally, the difference in location of F127 in the open and closed states was calculated, showing that although F127 moves between the two states, it does not move away from the lipid headgroups [46].

### MscS Gating Implications

Ec-MscS gates in response to applied tension overcoming the intrinsic bilayer tension allowing the channel to spring into the open state. Based on the structural models of the closed and open states, TM3B undergoes a twisting motion as the channel attains the open state. Once the channel has achieved the open state, protein-protein contacts stabilize an open state (Fig 6), which is maintained when tension is held steady as well as when additional tension to open MscL is applied. In this stable open state, the conductance of the channel does not change and the number of open channels remains relatively consistent.

As several non-conserved point mutations, S114I, L123F, F127, and A120S, within TM3B illustrate, the addition of significant bulk to the protein disturbs the packing arrangement that destabilizes the stable open state, allowing the channel to rapidly achieve an inactive state, as is normally only observed at low pH. The majority of these channels open to a full conductance, but either inactivate once tension is applied, or appear to flicker back and forth between the closed and open states. In the case of L123F, the destabilization of the stable open state leads to channels that flicker back and forth as long as tension is applied and do not appear to inactivate at any point. The motion of TM3B from the closed to open state is not typically perturbed by these mutations as shown by the wildtype pressure threshold ratios and conductances observed for these point mutations. However, in the majority of these point mutations, the stability of the full open state has been altered.

Some of these residues, for example F127, are close to the predicted lipid head groups, others are potentially within the hydrophobic lipid tails, such as L118 (Fig 6A and 6C) [9, 21, 22, 46]. It is potentially interesting that F127 shows proximity to the lipid headgroups, as the long-range interaction could be with the charged headgroup or potentially facilitated by the lipids. L118, based on structures in the open and closed states, is potentially exposed to the lipid tails and alterations within this region could potentially destabilize this interaction (Fig 6C). However, as Ec-MscS gates like a Jack-in-the-Box, this destabilization of the lipid interactions would be predicted to be a slight gain-of-function mutation, which is observed for L118F. This residue is not predicted to directly interact with the lipids, however the expansion in the tightly packed TM3B region leads to a slight destabilization of the lateral tension applied by the lipids. None of the remaining residues, S114, A120, and L123, are predicted to be on the surface of the protein based on the closed and open structures (Fig 6A and 6B). Our results confirm that the interactions of TM3B are essential for rate at which Ec-MscS enters the inactivated state as suggested by Koprowski et. al. and Rowe et. al. [27, 28]. Together these results confirm that TM3B is tightly packed in the open state and the inactivated states and that small alterations within this region lead to detectable kinetic differences.

## Conclusion

The gating motions that Ec-MscS undergoes in response to tension applied to the membrane are thought to be conserved throughout the MscS superfamily. The high conservation within TM3B for the superfamily suggests that this region plays an important role in the gating cycle for these channels. By making point mutations within Ec-MscS we have determined that one role for this region is stabilization of the full open state, and that even small changes to these residues leads to channels that can rapidly inactivate. Members of the MscS superfamily that are mechanosensitive do not show rapid inactivation in response to tension in the membrane when studied at neutral pH [47–49]. However, it now appears that the inactivation observed at low pH in Ec-MscS could be due in part to protein-protein contacts formed by residues in TM3B, suggesting that gating kinetics throughout the MscS superfamily are potentially unique to each subfamily. However, it should be noted that for the majority of the superfamily channels studied, the TM3B sequence, studied in this manuscript, is not an identical match for Ec-MscS, but instead often contain several of the highly conserved superfamily residues. These results suggest that this region in TM3B is dynamic in the gating cycle and that impairment of the formation of the protein-protein contacts required for stability of the full open state can lead to drastically different kinetic phenotypes for Ec-MscS and may explain some of the kinetic differences observed within the MscS superfamily.
